# Effects of Endogenous Non-Starch Nutrients in Acorn (*Quercus wutaishanica* Blume) Kernels on the Physicochemical Properties and In Vitro Digestibility of Starch

**DOI:** 10.3390/foods11060825

**Published:** 2022-03-14

**Authors:** Mohe He, Tianyi Ding, Yanwen Wu, Jie Ouyang

**Affiliations:** 1 Department of Food Science and Engineering, College of Biological Sciences and Technology, Beijing Key Laboratory of Forest Food Process and Safety, Beijing Forestry University, Beijing 100083, China; 18810657255@163.com (M.H.); 15624954327@163.com (T.D.); 2 Institute of Analysis and Testing, Beijing Academy of Science and Technology (Beijing Center for Physical and Chemical Analysis), Beijing 100089, China; wu_yanwen@163.com

**Keywords:** acorn, starch, protein, lipid, β-glucan, physicochemical properties

## Abstract

The present study investigated the multi-scale structure of starch derived from acorn kernels and the effects of the non-starch nutrients on the physicochemical properties and in vitro digestibility of starch. The average polymerization degree of acorn starch was 27.3, and the apparent amylose content was 31.4%. The crystal structure remained as C-type but the relative crystallinity of acorn flour decreased from 26.55% to 25.13%, 25.86% and 26.29% after the treatments of degreasing, deproteinization, and the removal of β-glucan, respectively. After the above treatments, the conclusion temperature of acorn flour decreased and had a significant positive correlation with the decrease in the crystallinity. The aggregation between starch granules, and the interactions between starch granules and both proteins and lipids, reduced significantly after degreasing and deproteinization treatments. The endogenous protein, fat, and β-glucan played key roles in reducing the digestibility of acorn starch relative to other compounds, which was dictated by the ability for these compounds to form complexes with starch and inhibit hydrolysis.

## 1. Introduction

Acorn is a general name for the fruit of the *Fagaceae* family of oak trees and their close relatives, which are widely distributed throughout the northern hemisphere. There are seven genera and 900 species within the Fagaceae throughout the world, including *Fagus*, *Castanea*, *Castanopsis*, *Lithocarpus*, *Trigonobalanus*, *Quercus*, and *Cyclobalanopsis*. Acorns have a long history of being used as a raw material in staple foods in Northern China, with an annual harvest in China of 6–7 million tons [1]. Fresh acorn contains about 42% moisture, 49% starch, 4.2% protein, 5.2% fat, 1.7% ash and 2.7% fibre in several *Quercus* species [2]. In addition, a total of 12 fatty acids have been detected in acorns, among which linolenic acid, linoleic acid, and palmitic acid were the most abundant. Moreover, 17 amino acids, including five essential amino acids, have also been detected in acorns [3,4]. Therefore, acorns have good potential as an alternative functional food and their by-products are also valuable [5].

Starch is the major component of acorns and acorn starch granules are spherical or ovoid with a diameter of 3–17 μm, exhibit the A-type X-ray diffraction pattern, and have a comparable weight-average molar mass of 3.9 × 10^8^ g/mol and an apparent amylose content (AAC) of 43.4% [1]. The digestibility of starch is crucial in determining the health benefits of acorn products [6]. Based on the digestion time, starch can be divided into either rapidly digested starch (RDS), slowly digested starch (SDS), and resistant starch (RS) [7]. The factors that affect starch digestibility include internal factors, such as the molecular structure, granule size, and crystal type, and external ones, such as processing methods and physical or chemical modifications [8]. Endogenous protein, fat, and other non-starch components may form macromolecular complexes with starch on the surface of the starch granules, which shows great influence on the digestibility of starch [9,10]. The digestion of starch is a complex enzymatic hydrolysis process, including enzyme diffusion, absorption, and catalytic event [11]. Proteins are found to reduce starch hydrolysis by interacting with starch or by limiting the access of enzyme diffusion [12]. The existence of surface lipids will increase hydrophobicity, and then affect water absorption, swelling and amylose leaching during processing [13]. In addition, lipid is the bridge between protein and starch as its functional carboxyl group is essential for the complex formation [14]. The existence of β-glucan in carbohydrate foods was found to favour a reduction in the rate of starch digestion, thus to maintain a low glycemic index (GI) [10]. The foods with low GIs can stabilize blood glucose, and potentially prevent obesity and cardiovascular diseases [15].

A large number of studies have reported how the endogenous proteins and lipids affected the properties of some kinds of starches, e.g., wheat starch [16], rice starch [17], naked oat starch [9], barely starch [18]. However, to the best of our knowledge, few studies have focused on similar research on acorns. After the removal of non-starch components, starch granules may undergo two significant changes. One is the change of the microstructure and the surrounding environment of starch granules; another one is the contact path and mode of interaction between starch granules and enzymes [18]. Therefore, the present study aimed to explore the physicochemical properties of acorn starch (AS) and the digestibility of acorn flour after the removal of fat, protein, or β-glucan. We hypothesized that the physicochemical properties of acorn flour would change significantly after the removal of non-starch components and that the presence of proteins and lipids on the surface of the AS would result in a decrease in the in vitro digestibility of starch. The results of this study would be helpful for understanding the influence of the non-starch nutrients in acorn kernels on the digestibility of starch.

## 2. Materials and Methods

### 2.1. Materials and Chemicals

*Quercus wutaishanica* Bl. was harvested in Yanji, Jilin, China. Three batches of fresh acorn fruits were shelled and cut into small pieces of 0.5 cm in length before being pulverized, dried at 40 °C for 24 h, and passed through an 80-mesh sieve. The sieved powder was marked as acorn flour (AF). Isoamylase (1000 U/mL) from *Pseudomonas adaceae* was bought from Aoboxing Biotechnology Co., Ltd. (Beijing, China). Porcine pancreatic α-amylase (9 U/mg) and glucosidase from *Saccharomyces cerevisiae* (23.5 U/mg) were obtained from Yuanye Biotechnology Co., Ltd. (Shanghai, China). All other chemicals were purchased from Beijing Chemical Reagent Co. (Beijing, China).

### 2.2. Removal of Nutrients from Acorn Flour

#### 2.2.1. Preparation of Acorn Starch

Acorn flour (20 g) was soaked in 40 mL of a NaOH solution (0.2%, *w*/*v*) for 2 h, the resulting suspension was filtered through a filter paper, and the filtrate was discarded. The filter cake was treated repeatedly based on the above steps until the filtrate was a light-yellow colour. After scraping off the brown impurities on the upper layer of the filter cake, the solid residue was repeatedly washed with distilled water until the pH of the water was less than 8.0. Finally, the solid residue was dried at 40 °C for 24 h to obtain the AS [9].

#### 2.2.2. Preparation of Degreased Acorn Flour

Acorn flour (5 g) was mixed with 25 mL of petroleum ether, and the suspension was stirred at 25 °C for 15 min, and then centrifuged at 5000× *g* for 4 min. The supernatant was decanted, and 25 mL of petroleum ether was added to resuspend the pellet, then stirred as described above and centrifuged again. This degreasing procedure was repeated five times. The final pellet was dried at 40 °C for 24 h and marked as degreased acorn flour (DGAF) [9].

#### 2.2.3. Preparation of Deproteinized Acorn Flour

Acorn flour (20 g) was added to a mixture of alkaline protease (0.2 g, 200 U/mg) in 180 mL of carbonate buffer (0.02 mol/L, pH 9.0). The suspension was stirred at 45 °C for 1 h and centrifuged at 4000× *g* for 10 min. The pellet was hydrolysed by the same method, washed with distilled water until the pH of the water was neutral, and dried at 40 °C for 24 h to obtain the deproteinized acorn flour (DPAF) [19].

#### 2.2.4. Preparation of De-β-Glucan Acorn Flour

The method used for the removal of β-glucan from the AF was a modification of the method by Zhang, Luo, and Zhang [20]. β-glucanase (16 mg, 50 U/mg) was dissolved in 200 mL of sodium acetate buffer (0.1 mol/L, pH 5.2). After adding 20 g of the AF to the solution, the resulting mixture was stirred continuously at 55 °C for 45 min. The mixture was centrifuged at 4000× *g* for 10 min, and the supernatant was discarded. The pellet was dried at 40 °C for 24 h and marked as de-β-glucan acorn flour (DβAF).

### 2.3. Determination of the Proximate Nutritional Components and Amylose Content

The contents of protein, fat, total starch, and β-glucan were determined by the AACC 46–13.01, 30–25.01, 76–13.01, and 32–22.01 methods [21], respectively. The amylose content was determined using the K-AMYL 07/11 kit (Megazyme International, Wicklow, Ireland).

### 2.4. Determination of Molecular Weight of Starch

The distributions of molecular weight and chain length of the AS were determined by size-exclusion chromatography (SEC), with which the molecules were separated based on size or hydrodynamic volume [22]. A solution of starch (20 mg) in 4 mL of DMSO (90% in distilled water, *v*/*v*) was heated at 100 °C for 15 min and then cooled to 25 °C under continuous stirring for 24 h. Subsequently, the mixture was diluted with 20 mL of 95% (*v*/*v*) ethanol and centrifuged at 3500× *g* for 10 min. The supernatant was discarded, and the pellet was reconstituted in 4 mL of deionized water, and the resulting suspension was stirred in a water bath at 100 °C for 15 min. The mixture was cooled to 25 °C and filtered through a 5-μm sieve, and then the filtrate was passed through a gel-permeation chromatography column equipped with refractive index detector (RID-20, Shimadzu, Kyoto, Japan). The mobile phase was an aqueous solution of 0.06% NaN_3_ (ultrasonicated before injection), the flow rate was 0.2 mL/min and the SEC column (TSK gel GMPWXL, TOSOH, Tokyo, Japan) temperature was 60 °C. The ASTRA 6.1 software package (Wyatt Technology Inc., Goleta, CA, USA) was used to determine the molecular weight, from which the polydispersity index (D) was calculated based on the following equation:

D = Mw/Mn(1)

where Mw is the weight average molecular weight, and Mn is the number average molecular weight.

### 2.5. Chain Length Distribution (CLD) of Amylopectin

The extracted AS sample (10 mg) was suspended in 2 mL of 95% (*v*/*v*) ethanol, and the suspension was heated at 80 °C for 30 min and then centrifuged at 4000× *g* for 5 min. The pellet was washed with anhydrous ethanol twice, and the treated starch was suspended in 5.0 mL deionized water and heated at 100 °C for 1 h to obtain the gelatinized acorn starch. Subsequently, 50 mL of sodium acetate buffer (600 mmol/L, pH 4.6) and 10 mL of 2.0% (*w*/*v*) sodium azide were added to 1.0 mL of the gelatinized starch sample. Isoamylase (12 mL, 1000 U/mL) was added to the AS solution to enzymatically debranch the starch at 40 °C for 24 h. The debranched starch sample was neutralized with 0.1 mol/L NaOH and then lyophilized. The dried debranched starch was dissolved in DMSO/LiBr solution for SEC analysis, following the method of Kuang, Xu, Wang, Zhou, and Liu [23]. The degree of polymerization (DP) was calculated by the formula:

M = 162.2·(DP − 1) + 18.0(2)

where M is the molecular weight.

### 2.6. Scanning Electron Microscopy (SEM)

The starch samples were placed on an aluminium tape with double-sided transparent tape, sputtered with gold, and photographed at 6 k× using a scanning electron microscope with an accelerator potential of 10 kV (JSM-6700F, JEOL, Tokyo, Japan).

### 2.7. The Particle Size Distribution

The sizes of the starch particles were determined by a Mastersizer 3000 laser particle size analyser (Malvern Instruments Ltd., Malvern, UK) equipped with a 300 mm lens and operating in polydispersity analysis mode. Approximately 0.1 g of starch was suspended in 100 mL distilled water and mixed using a magnetic stirrer for 30 min at room temperature before measurement. The size distribution measurements were made at intervals of 2 min and was reported as an average after three repetitions. The results were expressed as ratios (%) of large (>25 μm), medium (10–25 μm), small (5–10 μm), and very small (<5 μm) particles [24].

### 2.8. X-ray Diffraction Pattern (XRD) Analysis

The XRD analysis was performed by an X-ray diffractometer (D8 ADVANCE, Bruker Corp., Karlsruhe, Germany) operating at 30.0 kV and 40.0 mA. The diffraction scan range (2θ) ranged from 5° to 45° with a scanning speed of 2°/min and the step width of 0.01. The relative crystallinity of the samples was calculated by the DIFFREAC. EVA V3.1 software based on the following equation [25]:

Relative crystallinity (%) = (Area under peaks/Total area) × 100(3)

### 2.9. Fourier Transform Infrared Spectroscopy (FT-IR) Analysis

The FT-IR spectra of the sample were acquired by a Tensor 27 FT-IR spectrometer (Bruker Corporation, Billerica, MA, USA). The pellets containing 1% (*w*/*w*) of the sample in KBr were prepared, and then was pressed into transparent disks. Spectra were acquired over the range of 4000–400 cm^−1^ (32 scans) with a resolution of 4 cm^−1^.

### 2.10. Thermodynamic Characteristic Analysis

The thermodynamic parameters of the samples were measured by differential scanning calorimetry (DSC 200F3, Netzsch, Germany). The sample (3 mg) and distilled water were mixed at a ratio of 1:3.5 (*w*/*v*), and the temperature range was set to 20–120 °C with a heating rate of 10 °C/min. The onset temperature (T_o_), peak temperature (T_p_), conclusion temperature (T_c_), and gelatinization enthalpy (∆H) were all obtained by analysing the DSC curve.

### 2.11. In Vitro Digestibility

The digestibility was assessed according to a method described by Flores, Singh, Kerr, Pegg, and Kong with some modifications [26]. Simulated gastric and intestinal juices were prepared according to the procedure reported by Zhang, Yang, Liu, and Ouyang [27]. The sample (600 mg) was mixed with 6 mL of the prepared simulated oral juice, and the mixture was shaken for 20 s. Then, 12 mL of the simulated gastric juice was added, and the resulting mixture was shaken at 180 g for 1 h. Subsequently, the simulated bile and intestinal juice were added, the pH was adjusted to 8.0, and the mixture was shaken at 37 °C and 150 rpm. Aliquots (0.5 mL) of the enzymatic hydrolysate were removed at different times (10, 20, 40, 60, 90, 120 and 180 min), and the enzyme was inactivated with anhydrous ethanol (4.5 mL). The resulting mixture was centrifuged at 8000× *g* for 5 min. Subsequently, the glucose content (Gt) was analysed by the method of 3,5-dinitrosalicylic acid (DNS). The hydrolysis rate was calculated according to the equation:

Hydrolysis rate (%) = Gt × 0.9/starch content (mg)(4)

where Gt was the glucose content (mg). The ratios of RDS, SDS, and RS were calculated according to the formulas:

RDS (%) = (G_20_ − FG) × 0.9/TS (5)

SDS (%) = (G_120_ − G_20_) × 0.9/TS (6)

RS (%) = [TS − (RDS + SDS)]/TS(7)

where G_20_ and G_120_ were glucose content (mg) after enzymatic hydrolysis for 20 min and 120 min, respectively, FG was the content of free glucose in the starch before enzymatic hydrolysis (mg), and TS was the total starch content in the sample (mg).

### 2.12. Statistical Analysis

Each sample was measured in triplicate. The SPSS 23.0 software (IBM Corp., Armonk, NY, USA) was used for the analysis of variance (ANOVA) of the experimental data, Duncan’s Multiple Range Test was used to determine the significant difference level (*p* < 0.05) between the average values of each group, and Pearson’s test was used to analyse the correlation of the statistical data.

## 3. Results

### 3.1. Proximate Nutritional Components and Starch Molecular Structure

The contents of moisture, total starch, lipids, protein, and β-glucan in the AF were 6.2%, 58.3%, 5.9%, 8.4% and 6.8%, respectively (Table 1). The total AS content was lower than the starch content in corn (72.6%), wheat (68.1%), cassava (80.0%), potato (80.0%), and rice (85.0%) [13,28,29,30]. In previous studies, the total starch content in acorns ranged from 31.4% to 58.0% [2,5,6]. The difference in growing regions, harvest times, and detection methods could be responsible for the variation in the starch content in the same variety of acorn. The amylose content in *Q. wutaishanica* measured in the present study was 33.1% of starch, which was similar to one previous report [1] and lower than the amylose content in peas (41.1%), lentils (38.0%), and broad beans (39.9%) [31].

The Mw of the starch from *Q. wutaishanica* acorns was 4.02 × 10^8^ g/mol, and the Mn was 2.17 × 10^8^ g/mol, from which a polydispersity index of 1.85 was calculated. Meanwhile, the Mw of the starch from *Q. palustris* acorns was 3.93 × 10^8^ g/mol, and the Mw/Mn was 1.70, both of which were similar to the values determined in the present study. The Mw of the starch from *Q. wutaishanica* acorns was higher than the Mw values of the starch from potato (1.7 × 10^8^ g/mol) and wheat (3.1 × 10^8^ g/mol) [1]. The polydispersity index of AS was significantly lower than the index values of starch from other plants, such as water chestnuts (58.4), wheat (11.3), and corn (2.9) [32]. The correlation between the polydispersity index and the crystal form of starch indicated that the polydispersity index of A-type crystals was generally higher than that of B-type crystals [32]. The extremely low polydispersity index of AS may have advantages in the processing and utilization of acorns [1].

The chains of amylopectin can be classified based on the DP: chain A (DP 6–12), chain B1 (DP 13–24), chain B2 (DP 25–36), and chain B3 (DP ≥ 37) [33]. The chains A, B1, B2, and B3 of AS had CLDs of 17.2%, 45.3%, 17.0% and 18.5% (Figure S1), respectively, with an average chain length of 27.3, which was higher than the DP of the starch amylopectin derived from acorns of *Q. palustris* (DP of 25.5) [1]. Short-chain starches (DP 6–12) are unfavourable for the formation of secondary crystalline structures [28], while chains with DP ≥ 37 easily form crystals, which may lead to higher gelatinization temperatures and lower digestibility [34].

### 3.2. Proximate Constituents of AF after Removing Non-Starch Nutrients

For treatment, AF samples were separately degreased, deproteinized, and rid of β-glucan. The lipid removal rate to prepare the DGAF was 81.4%, and the β-glucan removal rate to prepare the DβAF reached 83.8%. For the DPAF, the protein content decreased from 8.4 to 2.0% after deproteinization. Lipids and protein were mostly removed from the AS, which was consistent with the results by Tang et al. [9] and Annor et al. [35].

Different treatment methods affected the amylose content in the AF, with the order of amylose content being DPAF > AF > DGAF > AS > DβAF. The significantly (*p* < 0.05) decreased amylose content in DβAF could be attributed to amylose leaching [36]. The increased amylose content of the DPAF might have been attributed to the hydrolysis of starch-protein complexes, enabling branches with long chain lengths to be exposed after the unwinding of amylopectin, which also showed similar properties compared to amylose in the determination of amylose content [17,37].

### 3.3. Changes of Microstructure and Particle Size

**Figure 1:**
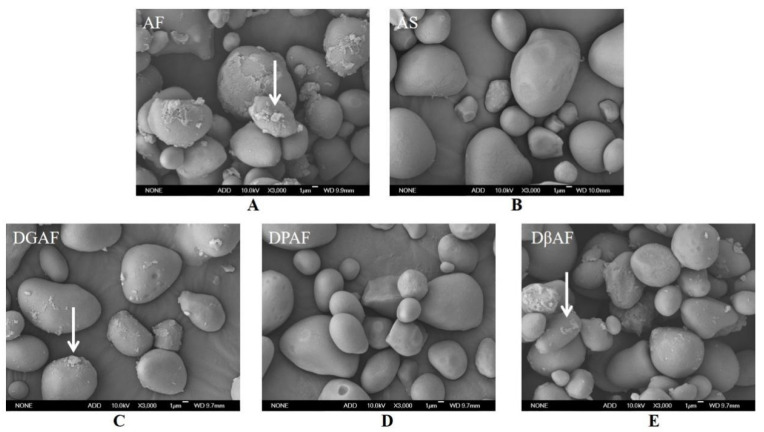
SEM images of AF and after removal of non-starch nutrients (×3000). (**A**) AF, acorn flour; (**B**) AS, acorn starch; (**C**) DGAF, degreased acorn flour; (**D**) DPAF, deproteinized acorn flour; (**E**) DβAF, de-β-glucan acorn flour.

The SEM images of AF and after degreasing, deproteinization, and de-β-glucan were shown in Figure 1. The shapes of the AS were oval, round, kidney-shaped, or irregular polygon, and were consistent with previous reports [5]. As mentioned in two different studies by Yang et al. [37] and Ye et al. [19], there were also many small particles attached to the surface of the starch particles, and aggregation was observed in the small particles. After degreasing, the aggregation of the small particles disappeared, and the sharp edges and corners increased, while the small particles still adhered to the surface of the starch granules (Figure 1C). This may be attributed to the low lipid level in AF, and the removed free fatty acid acted as a conjugate between proteins, starch, and β-glucan, which was consistent with the previous reports [18,19,37]. After treatment of the AF to remove β-glucan, the aggregation of starch particles was still observed, and the SEM image was smoother than the untreated AF (Figure 1E). It appeared that the small particles attached to the surface of the starch particles were proteins because the SEM micrographs of the DPAF (Figure 1D) and AS (Figure 1B) were smooth, but the micrographs of the DGAF (Figure 1C) and DβAF (Figure 1E) showed that the surfaces of the starch granules from these treated flour samples still contained many small particles. Yang et al. [37] and Ye et al. [19] also found that, after deproteinization, the surface of the particles became smooth, and the small particles had disappeared. Ye et al. [19] used fluorescent dyes to visualize the positions of proteins and observed that the protein particles were mostly featured on the surface of the starch particles.

To further analyse the changes in particle size, the starch granules were divided into two size ranges: large (>10 µm) and small (<10 µm). Compared to AF, the proportion of starch granules with sizes of <10 µm in the DGAF sample increased, but granules with sizes of >10 µm decreased (Table 2). It was previously determined that the average starch granule size in buckwheat flour decreased from 90.05 to 80.64 µm after degreasing, which was attributed to the lipids on the starch surface [37]. It is noteworthy that the proportion of large granules of both AS and DβAF increased compared to AF, which may be attributed to the fact that removing non-starch components is beneficial to the formation of starch aggregates and starch-lipid-protein complexes [38]. After degreasing and deproteinization, a decrease in large starch granules and increase in small granules were observed, which was mainly ascribed to the fact that partial large-sized starch granules were converted to small particles [18].

### 3.4. Long- and Short-Range Molecular Order

**Figure 2:**
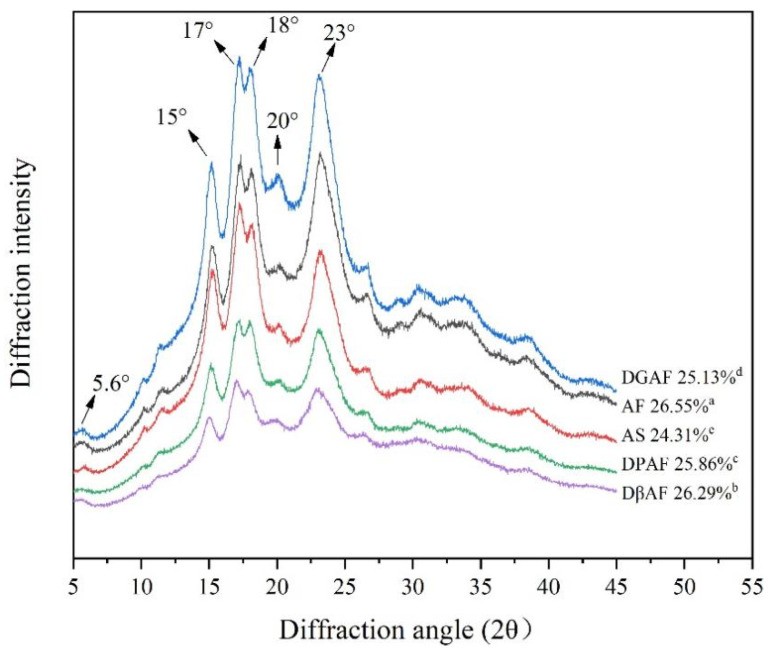
X-ray diffraction pattern of AF and after removal of non-starch nutrients. DGAF, degreased acorn flour; AF, acorn flour; AS, acorn starch; DPAF, deproteinized acorn flour; DβAF, de-β-glucan acorn flour. The different superscripts denoted significant differences within the same column (*p* < 0.05).

The XRD patterns of the acorn starch samples were shown in Figure 2. All samples had characteristic peaks at 5.6°, 15°, 17°, 18°, 20° and 23°, which indicated that the starch had remained as C-type crystals after degreasing, deproteinization, de-β-glucan, and starch extraction. This was consistent with a study by Li et al. [39] that also demonstrated that the crystal forms of both A-type and B-type starch granules were maintained after degreasing treatment. The relative crystallinity of the AF was determined to be 26.55%, which indicated a long-range molecular order. After different treatments, all the relative crystallinities decreased compared to AF, among which AS (24.31%) was the lowest, while the β-glucan removal treatment had the most trivial influence on the crystallinity (26.29%), and the crystallinity of the DGAF was in between (25.13%) the other two treated samples. Qin et al. [40] also indicated that the physical arrangement of the starch chains in amorphous starch granules and in the crystalline regions changed greatly after degreasing or deproteinization, which led to the decrease in the relative crystallinity.

The XRD peak at 2θ = 20° was the characteristic peak of the V-shaped crystal, which represented the existence of an amylose-lipid complex in the granular starch [41]. It was observed that the characteristic peak of 2θ = 20° still existed in the DGAF, revealing that the amylose-lipid complex still existed after degreasing. It also implied that degreasing treatment could not remove the lipids bound to the amylose. Li et al. [39] reported that the degreasing treatment may not remove the lipids inside the starch granules.

**Figure 3:**
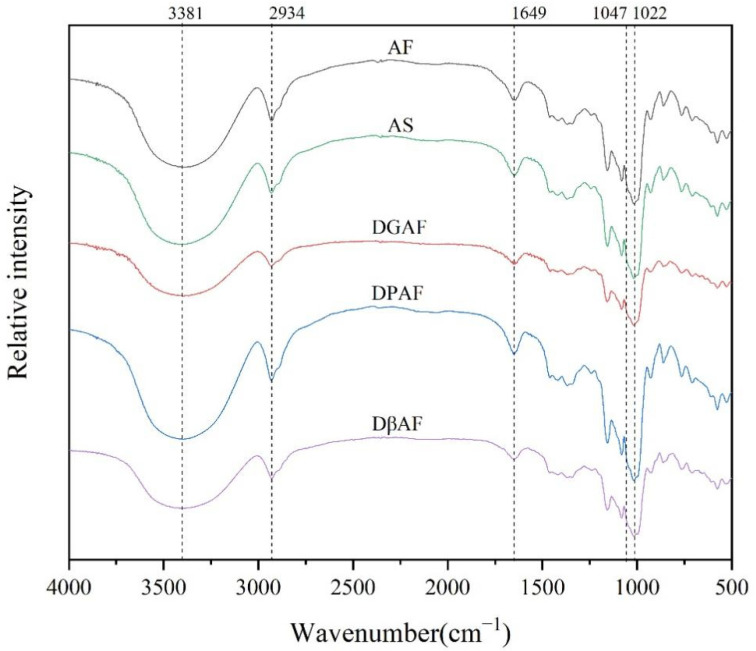
FT-IR spectra of AF and after removal of non-starch nutrients. AF, acorn flour; AS, acorn starch; DGAF, degreased acorn flour; DPAF, deproteinized acorn flour; DβAF, de-β-glucan acorn flour.

The FT-IR spectra of the different samples were similar (Figure 3). The peaks below 800 cm^−1^ corresponded to the skeleton mode vibration of the glucopyranose ring, and the peaks from 800 to 1500 cm^−1^ reflected the stretching vibrations of the glucose molecules. The characteristic peak at 1050–1100 cm^−1^ was attributed to the stretching of the C-O bond, and the peak at 1162 cm^−1^ was the characteristic peak for the C-O and C-C stretching vibrations of the dehydrated glucose ring. The peak at 1649 cm^−1^ represented the O-H vibration of water absorbed in the amorphous region of the starch granules. The peaks between 2800 and 3000 cm^−1^ demonstrated the C-H stretching mode, and the peaks observed in the range of 3000–3600 cm^−1^ were attributed to the starch. The peaks at 2934 cm^−1^ and 3381 cm^− 1^ represented the C-H deformation within the glucose unit and the O-H bond stretching vibrations, respectively [42].

The peak at 1022 cm^−1^ was the characteristic peak of the amorphous region of the starch granules, the peak at 1047 cm^−1^ was the characteristic peak of the crystalline region of the starch granules, and the ratio of the 1047 cm^−1^/1022 cm^−1^ peaks corroborated a short-range molecular order [43]. The ratio of 1047 cm^−1^/1022 cm^−1^ of the AF sample was 1.025, and this value increased after the removal of the non-starch components (Table 2). This might have been due to the disruption of the parallel alignment of the crystals by the different treatments [44]. After degreasing, the increase in the short-range molecular order might have been attributed to the starch-lipid complex formed from the localized interactions of the lipids and starch molecules, which destroyed the integrity of the starch molecules and exposed more short-chain molecules after degreasing [19,37].

### 3.5. Thermal Properties

**Figure 4:**
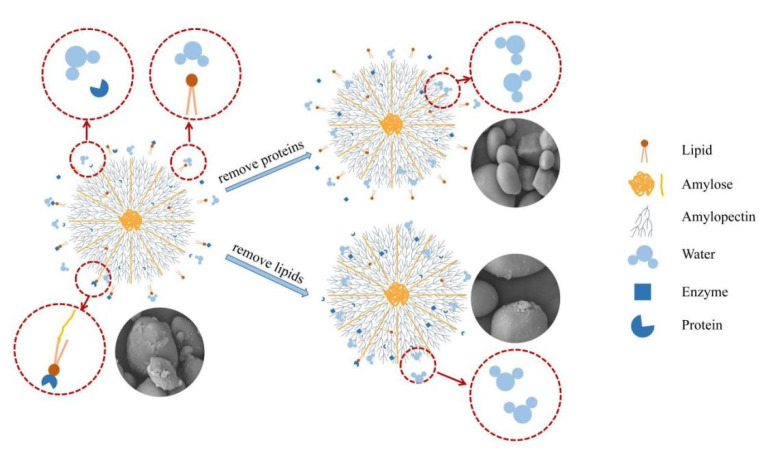
Schematic diagram of removing proteins and lipids from starch granule.

The T_o_ of the acorn starch after degreasing, deproteinization, de-β-glucan, and extraction (AS) all decreased relative to the untreated AF, with the T_o_ values following the order of AF > DGAF > DβAF > DPAF > AS (Table 3). The DSC results showed that the thermal stability of the AF decreased after the various treatments, which was consistent with previous experimental results [19,37]. There were two reasons for this change. First, the long-range and short-range molecular orders of the sample decreased, which contributed to the decrease in the stability of the samples. Second, the binding of lipids and proteins to the surface of the starch granules prevented the contact between water and the starch molecules (Figure 4); however, when the lipids and proteins were removed, the surface area of the starch granules increased enabling more contact between the water and starch and water molecules to more easily penetrate the starch granules [39,45].

There was a positive correlation (*r* = 0.724, *p* ˂ 0.05) between the relative crystallinity and the conclusion temperature (T_c_), which was consistent with the results from Wang, Liu, and Wang [46], because the ordered crystalline structure required the absorption of more heat compared to the amorphous region when it was destroyed.

### 3.6. In Vitro Digestibility

The hydrolysis rates of the acorn samples followed the order of AS > DGAF > DPAF > DβAF > AF (Figure S2). The AS, DGAF, and DPAF samples showed similar tendencies to undergo rapid hydrolysis during the first 20 min of digestion. The hydrolysis rates of these three samples slowed down between 20 and 120 min, while the AF and DβAF samples demonstrated a sharp increase in their hydrolysis rates between 20 and 40 min. It could be concluded that the non-starch components in the AS had significant effects on the digestibility of the starch. The order of the RDS content in the acorn samples was AS (17.2%) > DGAF (11.9%) > DPAF (9.2%) > DβAF (6.5%) > AF (4.9%), and the order of the RS content in the acorn samples was AF (69.2%) > DβAF (66.9%) > DPAF (64.0%) > DGAF (59.7%) > AS (59.0%) (Table 3). Li et al. [39] measured the digestibility of different starch granules after fat removal and found that the starch digestibility of both A-type and B-type granules were inhibited by lipids, but the inhibition was more apparent in the A-type granules.

When the protein and lipids on the surface of starch particles were removed, the corresponding increase in the free surface area and the exposure of enzyme binding sites accelerated the hydrolysis of the starch (Figure 4). The ability for the lipids to mitigate hydrolysis was stronger compared to the effect by proteins on the surface, even though the lipid content was lower than the protein content. However, lipids were able to form complexes with amylose, and the amylose-lipid complexes demonstrated a stronger resistance to hydrolysis compared to just amylose [10]. The ability of the lipids to inhibit starch hydrolysis was also stronger than the ability for proteins to inhibit hydrolysis in rice [19]. The inhibition mechanism of the β-glucan on the digestibility (hydrolysis) of starch not only involved the wrapping of the starch granules to reduce the surface area with which hydrolases and water could make contact, but also involved the competition of the β-glucan with water for the starch through the formation of noncovalent interactions between the β-glucan and starch molecules, thereby decreasing the localized concentration of water that is required for hydrolysis [45]. A study by Tang et al. [9] found that β-glucan had the greatest influence on starch digestibility compared to lipids and protein in naked oat starch. Because the non-starch components showed inhibitory effects on the digestibility of starch, people with chronic diseases, such as type-II diabetes, should incorporate more whole grain foods into their diets instead of refined food [10].

The RS was negatively correlated with amylose content (*r* = −0.691, *p* ˂ 0.05), which was attributed to the fact that amylose existed predominantly in the amorphous region. In addition, RS showed a significant negative correlation with ΔH (*r* = –0.783, *p* ˂ 0.01) and transition temperature (T_c_–T_o_) (*r* = –0.864, *p* ˂ 0.01), while RDS had a significant positive correlation with ΔH (*r* = 0.807, *p* ˂ 0.01) and transition temperature (*r* = 0.873, *p* ˂ 0.01). However, there was no significant correlation between relative crystallinity and digestibility, and the same conclusion was found in the experiments by Htoon et al. [47] and Chen et al. [48].

## 4. Conclusions

Acorn flour had a total starch content of 58.3%, and the amylose content ratio in starch was 33.1%. The average chain length (DP) of amylopectin was 27.3, and chain B2 accounted for 45.3%. From the SEM images, the particles attached to the surface of the starch granules were most likely protein molecules because the DGAF and DβAF starch samples had rough surfaces, while the surfaces of the AS and DPAF starch samples were smooth. The particle sizes of the starch granules decreased after degreasing. The different treatments performed on the AF did not change the crystalline structure of starch, but the relative crystallinity decreased after removing the non-starch components. The short-range order increased after the removal of non-starch components because of the tight interactions between the lipid molecules and starch molecules. In addition, RS had a significant negative correlation with ΔH and amylose content. Not only did these results provide further understanding of the low digestibility of acorn flour, but also, they provided evidence for the justification of acorns being used in various industrial, food-related applications, such as the development of acorn-containing food, which is beneficial for researching potential food alternatives for type II diabetics and people with diseases related to deficiencies in the metabolism of carbohydrates.

## Figures and Tables

**Table 1:** Proximate composition of AF and removal of non-starch nutrients.

Sample	Total Starch (g/100 g)	Amylose (% of Starch)	Lipid (g/100 g)	Protein (g/100 g)	β-Glucan (g/100 g)
AF	58.3 ± 2.9 ^d^	33.1 ± 2.3 ^a^	5.9 ± 0.1 ^a^	8.4 ± 0.2 ^ab^	6.8 ± 0.5 ^a^
AS	87.6 ± 2.2 ^a^	31.4 ± 2.1 ^ab^	0.9 ± 0.4 ^c^	1.6 ± 0.3 ^c^	2.4 ± 0.5 ^b^
DGAF	64.2 ± 3.1 ^bc^	32.6 ± 1.1 ^a^	1.1 ± 0.3 ^c^	10.2 ± 1.2 ^a^	6.4 ± 0.6 ^a^
DPAF	64.9 ± 1.9 ^c^	33.7 ± 3.2 ^a^	3.9 ± 0.2 ^b^	2.0 ± 0.5 ^c^	6.7 ± 0.9 ^a^
DβAF	67.7 ± 1.3 ^b^	29.8 ± 3.7 ^b^	6.5 ± 0.6 ^a^	10.7 ± 1.7 ^a^	1.1 ± 0.8 ^c^

The values are dry weight basis. The different superscripts denoted significant differences within the same column (*p* < 0.05). Abbreviations: AF, acorn flour; AS, acorn starch; DGAF, degreased acorn flour; DPAF, deproteinized acorn flour; DβAF, de-β-glucan acorn flour.

**Table 2:** Particle size distribution (%) and short-range molecular order of AF and after removal of non-starch nutrients.

Sample	˂5 μm	5–10 μm	10–25 μm	˃25 μm	IR Ratio of 1047 cm^−1^/1022 cm^−1^
AF	10.18	36.87	45.38	7.60	1.025 ± 0.023 ^d^
AS	10.56	34.50	40.84	14.14	1.049 ± 0.035 ^c^
DGAF	12.21	42.58	38.91	6.32	1.073 ± 0.004 ^b^
DPAF	10.89	39.33	35.45	14.32	1.077 ± 0.022 ^ab^
DβAF	9.27	34.36	40.18	16.20	1.081 ± 0.006 ^a^

The different superscripts denoted significant differences in the same column (*p* < 0.05). AF, acorn flour; AS, acorn starch; DGAF, degreased acorn flour; DPAF, deproteinized acorn flour; DβAF, de-β-glucan acorn flour.

**Table 3:** Thermal and digestive properties of AF and after removal of non-starch nutrients.

Sample	T_o_ (°C)	T_p_ (°C)	T_c_ (°C)	ΔH (J/g)	RDS (%)	SDS (%)	RS (%)
AF	66.3 ± 0.1 ^a^	72.7 ± 0.1 ^b^	81.6 ± 0.3 ^a^	3.3 ± 0.2 ^b^	4.9 ± 1.6 ^d^	25.9 ± 1.5 ^a^	69.2 ± 1.2 ^a^
AS	60.1 ± 0.4 ^d^	70.5 ± 0.2 ^a^	79.3 ± 0.2 ^b^	4.3 ± 0.2 ^a^	17.2 ± 2.0 ^a^	23.8 ± 2.1 ^b^	59.0 ± 2.4 ^c^
DGAF	65.1 ± 1.1 ^ab^	72.1 ± 0.3 ^b^	79.2 ± 0.1 ^b^	3.5 ± 0.3 ^ab^	11.9 ± 1.6 ^b^	28.4 ± 2.8 ^a^	59.7 ± 1.4 ^c^
DPAF	62.8 ± 0.4 ^c^	72.8 ± 0.9 ^b^	80.7 ± 0.4 ^a^	3.7 ± 0.1 ^ab^	9.2 ± 2.3 ^bc^	26.7 ± 1.5 ^ab^	64.0 ± 1.6 ^b^
DβAF	63.8 ± 0.2 ^bc^	72.0 ± 0.5 ^b^	80.8 ± 0.3 ^a^	3.3 ± 0.2 ^b^	6.5 ± 1.9 ^cd^	26.6 ± 2.6 ^ab^	66.9 ± 0.9 ^ab^

The different superscripts denoted significant differences within the same column (*p* < 0.05). AF, acorn flour; AS, acorn starch; DGAF, degreased acorn flour; DPAF, deproteinized acorn flour; DβAF, de-β-glucan acorn flour.

## Data Availability

Not applicable.

## Supplementary Materials

The following supporting information can be downloaded at https://www.mdpi.com/article/10.3390/foods11060825/s1, Figure S1: The branch-chain length distribution of acorn starch, Figure S2: Hydrolysis curve of the AF and after removal of non-starch nutrients.
