# Supplementary material for: Effects of Endogenous Non-Starch Nutrients in Acorn (Quercus wutaishanica Blume) Kernels on the Physicochemical Properties and In Vitro Digestibility of Starch
Source: Foods. 2022 Mar 14;11(6):825. doi: 10.3390/foods11060825 (PMC8947623; doi:10.3390/foods11060825)
Supplement: Supplementary file 1 [file foods-11-00825-s001.zip › foods-1639861-supplementary.pdf]

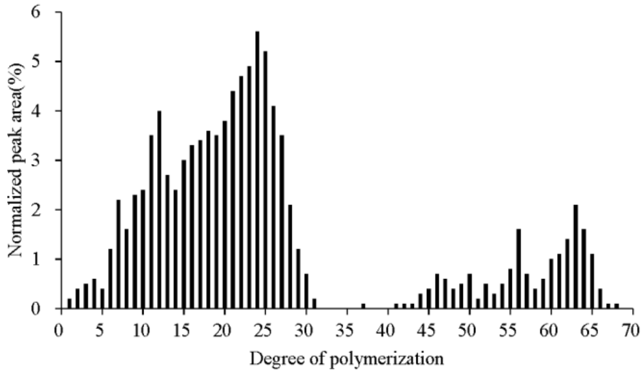

Figure S1. The branch-chain length distribution of acorn starch.

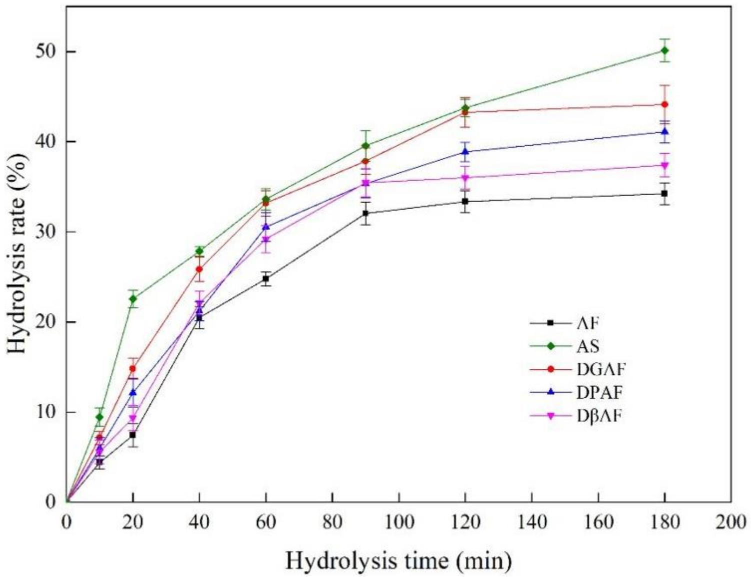

Figure S2. Hydrolysis curve of the AF and after removal of non-starch nutrients. Abbreviations: AF, acorn flour; AS, acorn starch; DGAF, degreased acorn flour; DPAF, deproteinized acorn flour; DβAF, de-β-glucan acorn flour.
